# A new formula for estimating fetal weight: The impression of biparietal diameter, abdominal circumference, mid-thigh soft tissue thickness and femoral length on birth weight

**Published:** 2013-11

**Authors:** Mojgan Kalantari, Arezou Negahdari, Shima Roknsharifi, Mostafa Qorbani

**Affiliations:** 1 *Department of Radiology, Mahdiyeh Hospital, Shahid Beheshti University of Medical Sciences, Tehran, Iran.*; 2 *Department of Public Health, Alborz University of Medical Sciences, Karaj, Iran.*; 3 *Non-Communicable Diseases Research Center, Endocrinology and Metabolism Population Sciences Institute, Tehran University of Medical Sciences, Tehran, Iran.*

**Keywords:** *Fetal weight*, *Soft tissue thickness*, *Abdominal circumference*, *Ultrasonography*, *Biparietal diameters*, *Femoral length*

## Abstract

**Background:** Abdominal circumference (AC), biparietal diameters (BPD) and femoral length (FL) are now the main parameters used to obtain estimated fetal weight (EFW). Although the role of soft tissue parameters in determining fetal weight was proved but clinical attention to mid-thigh soft tissue thickness (STT) is limited.

**Objective: **To find the impression of STT on birth weight (BW) and represent a new predictive formula.

**Materials and Methods:** One hundred and fourteen normal singleton term (36-42w) pregnancies with delivery within 72 hours were randomly selected to participate in this prospective cohort study. Variables measured by ultrasonography before birth included: AC, BPD, FL and STT. The actual neonatal BW was also measured after birth. Linear regression model was used and R square and p-value were reported.

**Results: **The mean (SD) of BW was 3406 (405) gr. R square was best fit for the model that STT was added to AC, BPD, FL (r^2^: 0.77). R square for the model using BPD, AC, FL and model using BPD, STT, FL was the same (r^2^: 0.7). Best fit formula was Log (BW)= 2.461+0.003BPD+0.001AC+0.007STT+0.005FL. AC (R: 0.67, p<0.001), STT (R: 0.50, p<0.001), BPD (R: 0.59, p<0.001), FL (R: 0.66, p<0.001) were significantly correlated with birth weight. AC had also significant correlation with STT (p=0.001)

**Conclusion:** This study showed adding STT to other variables in predictive models of fetal weight would provide a nice estimation (r^2^=0.77) and in cases that measuring AC is suboptimal STT may be a good replacement.

## Introduction

Birth weight is an important factor in delivery management. In extreme ranges of weight (<10^th^ and >90^th^ percentile) poor outcome is considerable. Higher birth weight is associated with both fetal and maternal complications (1-3). To estimate fetal weight, ultrasonography is the most common (1, 4). However the sensitivity and specificity does not have wide difference (12.6% and 92.1% for ultrasonography and 11.8% and 99.6% for clinical palpation respectively) (5). 

Different biometric variables obtained by ultrasonography such as biparietal diameter (BPD), abdominal circumference (AC), femoral length (FL), head circumference (HC) are used in different formulas to estimate fetal weight (EFW). These formulas have different levels of accuracy and in a systematic review none of them found to be preferred method in clinical practice due to the size of random errors (1, 6-8). Among the common used variables to determine fetal weight, AC has the fundamental correlation with birth weight. The correlation of AC with birth weight is 0.75 vs. 0.64, 0.67 and 0.55 for BPD, HC and FL respectively (1). The measurement of AC which is mostly representative of soft tissue mass in those formulas may be distorted by fetus condition (9, 10).

As the density of fat is 0.1 gr/mL less than average fetal density, a change in fat mass would definitely result in change of body weight (6). Several studies have shown the obvious effect of fat and lean masses as the main constrictors of fetal body on birth weight which might be of value as indicators of fetal growth and weight (9, 11-14).

Among different soft tissue variables of limbs that have been noticed to be in significant correlation with birth weight, mid-thigh soft tissue thickness (STT) is least worked on, however according to limited studies its role is probable (15-24). In addition, fewer studies were taken place in order to apply soft tissue parameters in predictive fetal weight formulas and finding the correlation of that with AC for distorted cases (24, 25). We have also found the gap of studies worked on fetal weight in Iranian population. The most noteworthy formula based on Iranian population is Honarvar formula that emphasizes on predicting fetal weight by single measurement of femoral length and there is no study on soft tissue parameters based on this population (7).

To fill these gaps, our study designed to find the impression of mid-thigh soft tissue thickness (STT) versus AC, BPD and FL on birth weight (BW). This study also provided new predictive formulas regarding above mentioned variables. 

## Materials and methods

This study was a prospective cohort study that was approved by ethics committee of Shahid Beheshti University of Medical Sciences, Tehran, Iran. One hundred and twenty five term (36-42w) pregnancies were randomly selected for this prospective study in 2010. Inclusion criteria were appointed as normal singleton pregnancy with delivery within 72 hours after evaluation. Therefore IUGR, macrosomia pregnancies, and pregnancies with polyhydramnios or oligohydramnios were excluded. By applying these criteria, 11 cases were excluded and finally 114 cases were included in this study after signing the informed consent.

**Figure 1:**
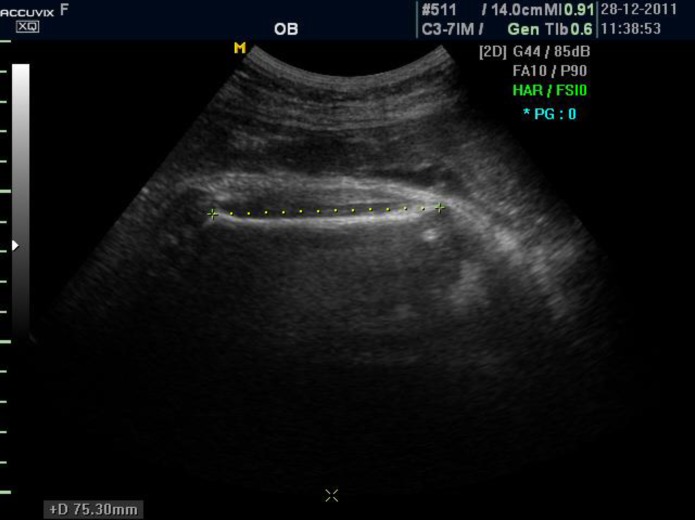
Appropriate section of femoral bone achieved when probe is parallel to the femoral shaft

**Figure 2:**
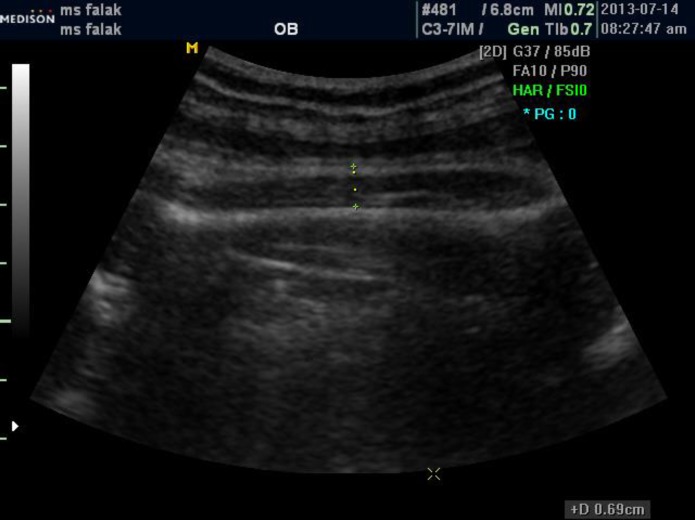
STT is defined as the distance between outer margins of skin to outer margin of femur shaft in the middle third of the thigh when magnified appropriate section is achieved

Ultrasonographic evaluation including AC, BPD, FL and STT were done for each case by two expert radiologists independently by using the unmodified Medison Accuvix (XQ) ultrasonography Machine with a 3.5 MHz probe. Then the mean of two results for each of those variables was applied for further analysis. AC, BPD, FL was assessed by standard methods (10). To measure STT (thickness of vastus lateralis muscle plus adipose tissue), appropriate section was achieved while probe was parallel to femoral bone (Figure 1), and then this section was magnified. STT was measured from outer margin of skin to outer margin of femur shaft in the middle third of the thigh (Figure 2). Actual birth weight (BW) also was measured immediately after birth.

**Statistical analysis**

To find the best formula that could predict the birth weight, different combination of variables including (AC, FL), (STT, FL), (BPD, AC, FL), (BPD, STT, FL) and (BPD, AC, STT, FL) were entered the linear regression model. Ultrasonic variables were used in millimeter (mm) unit as independent variables for synthesis of predicting formulas. Birth weight was measured in gram (gr) unit. Birth weight (BW) normality was tested by using Kolmogrov Smirnov test and due to lack of normality, log BW was considered as dependent variable. Data were analyzed in SPSS (Chicago, version 16). As an indicator of fitness of models for each combination of variables on birth weight R square was reported.

## Results

One hundred and fourteen cases participated in this study. Mean (SD) for maternal age was 27.1 (4.3) years and for gestational age of included pregnancies was 38.2 (1.2) weeks. Descriptive statistics regarding to variables of the study is shown in table I. AC (R: 0.67, p<0.001), STT (R: 0.50, p<0.001), BPD (R: 0.59, p<0.001), and FL (R: 0.66, p<0.001) were significantly correlated with birth weight. As well, STT was correlated with AC (R: 0.32, p=0.001). 

**Figure 3:**
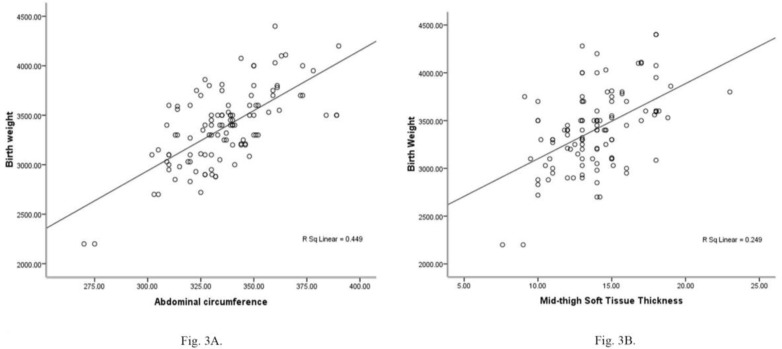
Scattered diagram for the impression of A: abdominal circumference on birth weight (R: 0.67, p<0.001), B: Mid-thigh soft tissue thickness on birth weight (R: 0.50, p<0.001).

Scattered diagrams for the impression of AC and STT on birth weight are shown in Figure 3A and Figure 3B respectively. Predictive birth weight formulas derived by linear regression model are illustrated in table II. The highest R square (0.77) was reported when STT was added to other ultrasound parameters. R square for the impression of AC along with BPD and FL was the same as STT in combination with those two variables (0.7).

**Table I:** Descriptive statistic of ultrasonic variables in term pregnancy (N= 114

**Variables**	**Mean**	**SD**	**Minimum**	**Maximum**
Biparietal diameter (mm)	92.20	3.057	86.00	101.00
Abdominal circumference (mm)	336.27	21.25	270.00	390.00
Soft tissue thickness (mm)	13.89	2.57	7.60	23.00
Femoral length (mm)	73.04	3.85	64.40	83.00
Birth weight (gr)	3406.32	405.42	2200.00	4400.00

**Table II:** Predictive formulas in different combination of variables and their predictive power (R square)

**Variables** *	**Predictive Formulas** †	**R Square**
AC, FL	Log (BW)=2.729+0.001AC+0.006FL	0.60
STT, FL	Log (BW)=2.878+0.007STT+0.008FL	0.53
BPD, AC, FL	Log (BW)=2.435+0.003BPD+0.001AC+0.006FL	0.69
BPD, STT, FL	Log (BW)=2.436+0.005BPD+0.009STT+0.007FL	0.70
BPD, AC, STT, FL	Log (BW)=2.461+0.003BPD+0.001AC+0.007STT+0.005FL	0.77

AC: Abdominal circumference, FL: Femoral length, STT: Mid-thigh soft tissue thickness, BPD: Biparietal diameter, BW: Birth weight.

* AC, BPD, FL, STT are obtained by ultrasound in millimeter unit.

† Unit of BW is grams.

## Discussion

The results of present study provided new formulas for estimating fetal weight. This study also showed that adding STT to other variables (BPD, AC and FL) in predictive models of fetal weight would provide the best estimation (r^2^=0.77) and the predictive strength of each formulas using STT or AC along with BPD and FL would be the same (r^2^=0.7).

Recent studies which tried to take account to limb soft tissue parameters in predictive formulas are few. Different limb soft tissue variables which were used to improve fetal weight estimation models includes thigh volume obtained by cross sectional images, fractional thigh volume, fractional limb volume, mid-thigh tissue area, mid-thigh soft tissue thickness and fetal abdominal subcutaneous tissue and thigh muscle and fat (19-25). Also those studies suggested new formulas by applying these variables (16, 19-24). Accuracy of some of these formulas was shown to be more than previous ones with less percentage of error (19-24). 

Larciprete *et al* and Scioscia studies, like our study emphasize on the impression of mid-thigh soft tissue thickness on birth weight (24, 25). The former study noted the significant improvement of birth weight prediction models when mid-thigh tissue area is added to other standard ultrasound variables. In this study this new formula had significantly lower error margin in comparison with other formulas (p<0.05) (25). Scioscia and his group designed a multi-phase study and found that STT is in high correlation with birth weight (p<0.001; r^2^=0.46). Then they recommend a new linear formula using STT and FL for estimating fetal weight. In phase3 of this study estimated fetal weight obtained by this new formula was shown to be highly correlated with actual birth weight (p<0.001; r^2^=0.68) (24).

In previously published models attention was concentrated toward diameters of head, abdomen and femoral bone (26-28). Among those variables, AC was shown to be of best predictive value (25, 29, 30). The value of AC highly depends on its correct measurement by considering some strict rules including location of spine at 3 or 9 o'clock of the transverse section, observing stomach at left site, existence of only one rib and the junction of the umbilical segment of left and right portal vein in the section (10, 11). 

All these rules may not be achievable all time. Therefore a question will be encountered: “Is there any good replacement for AC in the cases that we could not measure AC properly?” Recent studies showed that fetal fat amount is mostly correlated with fractional thigh volume (31, 32). Intellectually, because fetal weight is directly correlated with fetal fat amount, it would be mostly correlated with limb soft tissue than AC as well. Studies that specifically were designed to determine the accuracy of this hypothesis are rare, however some comparisons were made during other studies. Santolaya-Forgas *et al* and Balouet *et al* studies introduced soft tissue parameters superior to AC in predicting birth weight (14, 33). 

Scioscia study had shown that STT is significantly correlated with AC (p<0.001; r^2^=0.36) and both R squares for correlation of these variables with actual birth weight were about 0.46 (24). Compatible to those studies, our study showed significant correlation of AC with STT and also in regression of BW by BPD, STT, FL same R square was achieved as in model using BPD, AC, FL (r^2^=0.7). But contrary to abovementioned studies, we found higher R square in single measurement regression of BW by AC than STT (r^2^=0.449 vs. r^2^=0.249). Like our study, Larciprete *et al* study showed higher R square in single measurement regression of EFW by AC than mid-thigh tissue area (MTTA) (r^2^=0.59 vs. r^2^=0.19) (25). This study also mentioned that AC is correlated with MTTA (p<0.05). 

It would be minded that these results may not be completely reliable for replacement of AC by STT but it can open a window for further researches. Replacement of AC by STT seems to have some benefits. It could be of much use when positioning of the fetus makes the correct measurement of AC be distorted. Besides, FL section itself and linear measurement of STT in this section is much easier than measurement of AC, especially for non-expert operators. In addition ethnicity may play role in fetal weight, there are limited Birth Weight formulas based on Iranian population (7). Honarvar formula that has been shown to be accurate for Iranian population uses single measurement of femoral length (7, 8). Since soft tissue parameters have not been used in this formula, it is not comparable with our result. Short interval between ultrasonography measurement and birth weight, obtaining measurements by expert radiologists, finding linear formulas, making new windows for further researches are benefits of our study. The result of present study may be limited by two reasons: 1-The new formula is only applicable at term; 2-The accuracy of the new formula in comparison to others is unknown.

## Conclusion

In conclusion, our study emphasizes on adding STT to other ultrasound parameters in order to improving fetal weight prediction models and also suggest further researches on the subject of replacing AC by STT. We hope this can be useful in clinical practice especially when measurement of AC is distorted.

## Conflict of interest

None of the authors of this article had conflict of interest or any financial involvement that could affect the research.

## References

[1] Nahum GG Estimation of Fetal Weight. http://emedicine.medscape.com/article/%20262865-overview.

[2] Mocanu EV, Greene RA, Byrne BM, Turner MJ (2000). Obstetric and neonatal outcome of babies weighing more than 4.5 kg: an analysis by parity. Eur J Obstet Gynecol Reprod Biol.

[3] Jolly MC, Sebire NJ, Harris JP, Regan L, Robinson S (2003). Risk factors for macrosomia and its clinical consequences: a study of 350,311 pregnancies. Eur J Obstet Gynecol Reprod Biol.

[4] Peregrine E, O'brien P, Jauniaux E (2007). Clinical and ultrasound estimation of birth weight prior to induction of labor at term. Ultrasound Obstet Gynecol.

[5] Ashrafganjooei T, Naderi T, Eshrati B, Babapoor N (2010). Accuracy of ultrasound, clinical and maternal estimates of birth weight in term women. East Mediterr Health J.

[6] Dudley N (2005). A systematic review of the ultrasound estimation of fetal weight. Ultrasound Obstet Gynecol.

[7] Honarvar M, Allahyari M, Dehbashi S (2001). Assessment of fetal weight based on ultrasonic femur length after the second trimester. Int J Gynaecol Obstet.

[8] Firoozabadi RD, Ghasemi N, Firoozabadi MD (2007). Sonographic fetal weight estimation using femoral length: Honarvar equation. Ann Saudi Med.

[9] Balouet P, Speckel D, Herlicoviez M (1992). Ultrasonic estimation of fetal weight. Value of measuring limb fat. J Gynecol Obstet Biol Reprod.

[10] McGahan JP, Goldberg BB (2008). Diagnostic Ultrasound.

[11] Valensise H, Larciprete G, Arduini D, Lorenzo AD (2003). The fetal body compartments and their detection during pregnancy. A review. Acta Diabet.

[12] Lee W, Balasubramaniam M, Deter RL, Hassan SS, Gotsch F, Kusanovic JP (2009). Fractional limb volume-a soft tissue parameter of fetal body composition: validation, technical considerations and normal ranges during pregnancy. Ultrasound Obstet Gynecol.

[13] Catalano PM, Tyzbir ED, Allen SR, McBean JH, McAuliffe TL (1992). Evaluation of fetal growth by estimation of neonatal body composition. Obstet Gynecol.

[14] Balouet P, Hamel P, Domessent D, Allouche C, Speckel D, Barjot P (1994). [The estimation of fetal weight by measurement of the adipose tissue of the extremities. Use in the diagnosis of hypotrophy]. J Gynecol Obstet Biol Reprod (paris).

[15] Han Y, Lin H, Liu Y (1998). [Ultrasonic measurements of fetal thigh soft tissue thickness in the estimation of fetal weight]. Zhonghua Fu Chan Ke Za Zhi.

[16] Larciprete G, Valensise H, Barbati G, Di Pierro G, Jarvis S, Deaibess T (2007). Ultrasound‐determined fetal subcutaneous tissue thickness for a birthweight prediction model. J Obstet Gynaecol Res.

[17] Al-Hilli NMS (2009). Antepartum Detection of Macrosomic Fetus: Clinical Versus Sonographic, Including Humeral Soft Tissue Thickness. Med J Babylon.

[18] Schild R ( 2007). Three dimensional volumetry and fetal weight measurement. Ultrasound Obstet Gynecol.

[19] Lee W, Deter R, L Ebersole JD, Huang R, Blanckaert K, Romero R (2001). Birth weight prediction by three-dimensional ultrasonography: fractional limb volume. J Ultrasound Med.

[20] Song TB, Moore TR, Lee JY, Kim YH, Kim EK (2000). Fetal weight prediction by thigh volume measurement with three-dimensional ultrasonography. Obstet Gynecol.

[21] Srisantiroj N, Chanprapaph P, Komoltri C (2011). Fractional thigh volume by three-dimensional ultrasonography for birth weight prediction. J Med Assoc Thai.

[22] Lee W, Deter R, Sangi-Haghpeykar H, Yeo L, Romero R (2013). Prospective validation of fetal weight estimation using fractional limb volume. Ultrasound Obstet Gynecol.

[23] O'Connor C, Farah N, O'Higgins A, Segurado R, Fitzpatrick C, Turner MJ (2013). Longitudinal measurement of fetal thigh soft tissue parameters and its role in the prediction of birth weight. Prenat Diagn.

[24] Scioscia M, Scioscia F, Vimercati A, Caradonna F, Nardelli C, Pinto LR (2008). Estimation of fetal weight by measurement of fetal thigh soft‐tissue thickness in the late third trimester. Ultrasound Obstet Gynecol.

[25] Larciprete G, Di Pierro G, Barbati G, Deaibess T, Jarvis S, Valensise H ( 2008). Could birthweight prediction models be improved by adding fetal subcutaneous tissue thickness?. J Obstet Gynaecol Res.

[26] Scioscia M, Vimercati A, Ceci O, Vicino M, Selvaggi LE (2008). Estimation of Birth Weight by Two-Dimensional Ultrasonography: a critical appraisal of its accuracy. Obstet Gynecol.

[27] Kurmanavicius J, Burkhardt T, Wisser J, Huch R (2004). Ultrasonographic fetal weight estimation: accuracy of formulas and accuracy of examiners by birth weight from 500 to 5000 g. J Perinat Med.

[28] Melamed N, Yogev Y, Meizner I, Mashiach R, Bardin R, Ben-Haroush A ( 2009). Sonographic Fetal Weight Estimation Which Model Should Be Used?. J Ultrasound Med.

[29] Sokol RJ, Chik L, Dombrowski MP, Zador IE (2000). Correctly identifying the macrosomic fetus: improving ultrasonography-based prediction. Am J Obstet Gynecol.

[30] Dudley N, Chapman E (2002). The importance of quality management in fetal measurement. Ultrasound Obstet Gynecol.

[31] Lee W, Balasubramaniam M, Deter RL, Hassan SS, Gotsch F, Kusanovic JP (2009). Fetal growth parameters and birth weight: their relationship to neonatal body composition. Ultrasound Obstet Gynecol.

[32] Moyer-Mileur LJ, Slater H, Thomson JA, Mihalopoulos N, Byrne J, Varner MW (2009). Newborn adiposity measured by plethysmography is not predicted by late gestation two-dimensional ultrasound measures of fetal growth. J Nutr.

[33] Santolaya-Forgas J, Meyer WJ, Gauthier DW, Kahn D (1994). Intrapartum fetal subcutaneous tissue/femur length ratio: an ultrasonographic clue to fetal macrosomia. Am J Obstet Gynecol.

